# On the geometry of elementary flux modes

**DOI:** 10.1007/s00285-023-01982-w

**Published:** 2023-08-30

**Authors:** Frederik Wieder, Martin Henk, Alexander Bockmayr

**Affiliations:** 1 FB Mathematik und Informatik, Freie Universität Berlin, Arnimallee 6, 14195 Berlin, Germany; 2 Institut für Mathematik, Technische Universität Berlin, Straße des 17. Juni 136, 10623 Berlin, Germany

**Keywords:** Metabolic networks, Elementary flux modes, Steady-state flux cone, Face lattice, 92C42 Systems biology Networks, 52B05 Combinatorial properties of polytopes and polyhedra

## Abstract

Elementary flux modes (EFMs) play a prominent role in the constraint-based analysis of metabolic networks. They correspond to minimal functional units of the metabolic network at steady-state and as such have been studied for almost 30 years. The set of all EFMs in a metabolic network tends to be very large and may have exponential size in the number of reactions. Hence, there is a need to elucidate the structure of this set. Here we focus on geometric properties of EFMs. We analyze the distribution of EFMs in the face lattice of the steady-state flux cone of the metabolic network and show that EFMs in the relative interior of the cone occur only in very special cases. We introduce the concept of degree of an EFM as a measure how elementary it is and study the decomposition of flux vectors and EFMs depending on their degree. Geometric analysis can help to better understand the structure of the set of EFMs, which is important from both the mathematical and the biological viewpoint.

## Introduction

Constraint-based analysis of metabolic networks is an important area in computational biology (Bordbar et al. 2014; Fang et al. 2020). The stoichiometric and thermodynamic constraints that have to hold in a metabolic network at steady-state have led to the definition of the *steady-state flux cone*, which comprises all possible flux distributions over the network at steady-state.

An important concept to analyze the flux cone in a mathematically and biologically meaningful way are *elementary flux modes* (EFMs) (Schuster and Hilgetag 1994; Schuster et al. 2002), which provide an inner description of the flux cone consisting of a finite set of generating vectors (Gagneur and Klamt 2004; Wagner and Urbanczik 2005; Larhlimi and Bockmayr 2008; Jevremovic and Boley 2013). From a mathematical point of view, not all elementary flux modes are needed to describe the cone, i.e., the set of all EFMs does not form a minimal generating set, except for special cases (for example when all reactions are irreversible). Even for small networks, the number of elementary flux modes may be very large.

Larhlimi and Bockmayr (2009) introduced metabolic behaviors and studied outer descriptions of the flux cone based on *minimal metabolic behaviors* (MMBs), which are in a one-to-one correspondence with the minimal proper faces of the flux cone. Röhl and Bockmayr (2019) introduced the concept of a minimal set of elementary flux modes (MEMo) and gave an algorithm to compute such a set. A MEMo consists of an EFM from each minimal proper face of the flux cone together with a vector space basis of the lineality space. In general, a minimal proper face may contain many different EFMs.

The goal of this paper is to get a deeper understanding of the structure of the set of EFMs by further studying their geometric properties. We generalize the result of Larhlimi and Bockmayr (2009) and show that higher-dimensional faces of the flux cone can be characterized by metabolic behaviors. We study the distribution of EFMs over the faces of the flux cone and introduce as a measure of complexity the *degree* of a flux vector or EFM, which is the dimension of the inclusionwise minimal face containing it. The intuitive idea behind this is that the smaller the degree of a flux vector or EFM, the more elementary it is. More formally, we show that any flux vector of degree *k* can be decomposed into a sum of at most *k* EFMs of degree at most *k*. We prove upper bounds on the degree of EFMs and show that EFMs in the relative interior of the flux cone occur only in very special cases. We also study the effect of removing blocked reactions and redundant irreversibility constraints and deduce an upper bound on the cardinality of minimal metabolic behaviors.

## Polyhedral cones

In this section we introduce some mathematical background on polyhedral cones. For further reading we refer to Lauritzen (2013), Schneider (1993) and Schrijver (1986).

A vector $x \in \mathbb {R}^n$ is a *linear combination* of the vectors $x^1,\dots ,x^k \in \mathbb {R}^n$ if $x = \lambda _1 x^1 + \cdots + \lambda _k x^k,$ for some $\lambda _1,\dots ,\lambda _k \in \mathbb {R}$. If, in addition

$$\begin{aligned} \left\{ \begin{array}{ccc} \lambda _1,\dots ,\lambda _k \ge 0, \\ &{} \lambda _1 + \dots + \lambda _k = 1, \\ \lambda _1,\dots ,\lambda _k \ge 0, \; &{} \lambda _1 + \dots + \lambda _k = 1, \end{array} \right\} \text { we call~} x\hbox { a } \left\{ \begin{array}{ccc} \text {conic} \\ \text {affine} \\ \text {convex} \end{array} \right\} \text {combination.} \end{aligned}$$

For a nonempty subset $X \subseteq \mathbb {R}^n$, we denote by $\text {lin}(X)$ (resp. ${{\,\textrm{cone}\,}}(X)$, $\text {aff}(X)$, ${{\,\textrm{conv}\,}}(X)$) the *linear* (resp. *conic, affine, convex*) *hull of* *X*, i.e., the set of all linear (resp. conic, affine, convex) combinations of finitely many vectors of *X*.

A nonempty set $C \subseteq \mathbb {R}^n$ is a *convex cone*, if it is closed under conic combinations, i.e., $\lambda x + \mu y \in C,$ for all $x,y \in C$ and $\lambda , \mu \ge 0$. A convex cone *C* is *polyhedral* if it is the solution set of a system of finitely many homogeneous linear inequalities, i.e., if

$$\begin{aligned} C = \{x \in \mathbb {R}^n \mid Ax \ge 0\}, \end{aligned}$$

for some matrix $A \in \mathbb {R}^{m \times n}$. If a cone *C* is the conic hull of a finite set $X=\{x^1,...,x^s\} \subset \mathbb {R}^{n}$, it is called *finitely generated* and the set *X* is called a *generating set* of *C*. By the well-known theorem of Farkas-Minkowski-Weyl (see e.g. (Schrijver 1986)), a convex cone is polyhedral if and only if it is finitely generated. For the rest of this paper we will only consider polyhedral cones and often simply write cone.

If $C = \{ x \in \mathbb {R}^n \mid Ax \ge 0 \}$, is a polyhedral cone, an inequality $a x \ge 0$, where *a* denotes a row of *A* and *ax* the inner product of *a* and *x*, is called an *implicit equality in* $Ax \ge 0$, if $a x = 0$, for all $x \in C$. Following (Schrijver 1986), we denote by $A^= x \ge 0$ the system of implicit equalities in $Ax \ge 0$ and by $A^+ x \ge 0$ the remaining inequalities.

If removing an inequality $a x \ge 0$ from $Ax \ge 0$ does not change the associated cone *C*, the inequality is called *redundant*. If there are no redundant inequalities, the description $Ax \ge 0$ is called *irredundant*.

The *dimension* $\dim (C)$ of a cone *C* is the dimension of its affine hull $\text {aff}(C) = \{x \in \mathbb {R}^n \mid A^= x = 0\}$ and is equal to $n-{{\,\textrm{rank}\,}}(A^=)$. Since $0 \in C$, $\text {aff}(C)$ coincides with the linear hull $\text {lin}(C)$.

A vector $x \in C$ is in the *relative interior of* *C*, shortly $x \in {{\,\textrm{relint}\,}}(C)$, if there exists $\epsilon > 0$ such that $B_{\epsilon }(x) \cap \text {aff}(C) \subseteq C$, where $B_{\epsilon }(x)$ is the *n*-dimensional ball of radius $\epsilon$ centered at *x*. If $x \in C$ is not in the relative interior of *C*, it is in the *relative boundary of* *C*.

The *lineality space* of a cone $C = \{x \in \mathbb {R}^n \mid Ax \ge 0\}$ is given by ${{\,\mathrm{lin.space}\,}}(C) = \{x \in \mathbb {R}^n \mid Ax = 0\}$, which is the inclusionwise maximal linear subspace contained in *C*. A cone *C* is called *pointed* if its lineality space is trivial, i.e., ${{\,\mathrm{lin.space}\,}}(C) = \{0\}$. If a cone is pointed, it does not contain a line.

An inequality $a x \ge 0$ is called *valid* for *C* if $C \subseteq \{x \in \mathbb {R}^n \mid ax \ge 0\}$. A nonempty set $F \subseteq C$ is called a *face* of *C* if there exists an inequality $a x \ge 0$ valid for *C* such that $F = C \cap \{x \in C \mid ax = 0\}$. The hyperplane $\{x \in C \mid ax = 0 \}$ is then called a *supporting hyperplane* of *F*. Alternatively, a face can be characterized as a nonempty set $F \subseteq C$ with $F = \{ x \in C \mid A_{I,\star } x = 0\}$, where $A_{I,\star }$ is the submatrix of *A* whose rows belong to the set $I \subseteq \{1,\dots ,m\}$ (Schrijver 1986).

A polyhedral cone *C* has only finitely many faces, each face *F* of *C* is itself a polyhedral cone and $F' \subseteq F$ is a face of *F* if and only if $F'$ is a face of *C*. A *k*-dimensional face will also be called a *k*-*face*. A cone *C* is pointed if and only if it has a 0-face, namely the origin.

A face $F \ne C$ of *C* is called a *facet* if it is inclusionwise maximal, i.e., there is no other face $F' \ne C$ such that $F \subset F'$. If the description $Ax \ge 0$ of *C* is irredundant, there is a 1–1 correspondence between the facets of *C* and the inequalities in $A^+ x \ge 0$ (Schrijver 1986, Theor. 8.1). In particular, for every facet *F* there is an inequality $ax \ge 0$ from $A^+ x \ge 0$ such that $F = \{ x \in C \mid a x = 0 \}$. We have $\dim (F) = \dim (C) -1$ for every facet *F* of *C*, and every face of *C* (except *C* itself) is the intersection of facets of *C*.

The *face lattice* of a polyhedral cone *C* is the partially ordered set *L*(*C*) of all faces of *C*, partially ordered by set inclusion (Henk et al. 2017; Ziegler 1994). Two polyhedral cones $C,C'$ are *combinatorially equivalent* if there is a bijection from *L*(*C*) to $L(C')$ that preserves the inclusion relation. The *combinatorial type* of a polyhedral cone is the equivalence class under combinatorial equivalence.

### Proposition 2.1

Let $C = \{x \in \mathbb {R}^n \mid Ax \ge 0\}$ be a polyhedral cone and $z \in C$. Let $A_z^=$ be the submatrix of *A* whose rows correspond to the inequalities in $Ax \ge 0$ that are fulfilled with equality by *z*. Let *F* be the face of *C* defined by $F = \{x \in C \mid A_z^= x = 0\}$. Then

(i) *F* is the inclusionwise minimal face of *C* containing *z*,
(ii) $$\dim (F) = n - {{\,\textrm{rank}\,}}(A_z^=)$$,
(iii) $$z \in {{\,\textrm{relint}\,}}(F)$$.

### Proof

For $x \in \mathbb {R}^n$, define $I(x) = \{i \in \{1,\dots ,m\} \mid a_i x = 0\}$, where $a_i$ is the *i*-th row in *A*. Let $F' = \{x \in C \mid A_{I,\star }x = 0\}$ be a face of *C* containing *z*. Then $A_{I,\star }z = 0$ and thus $I \subseteq I(z)$, which implies $F = \{x \in C \mid A_{I(z),\star }x = 0\} \subseteq F'$ and statement (i) follows.

For $x \in F$, we have $I(z) \subseteq I(x)$. Therefore, *I*(*z*) has the minimal number of elements among *I*(*x*), where $x \in F$. The statements (ii) and (iii) now follow from Proposition 4.3 in Lauritzen (2013) and its proof. $\square$

If *C* is a cone with $\dim ({{\,\mathrm{lin.space}\,}}(C)) = t \ge 0$, a face of dimension $t+1$ is called a *minimal proper face* of *C*. For a pointed cone *C*, the minimal proper faces are the 1-faces, which are called the *extreme rays* of *C*. Equivalently, ${{\,\textrm{cone}\,}}(\{r\}) \subseteq C, r \ne 0,$ is an extreme ray of *C* if and only if $r = x + y$ implies $x,y \in {{\,\textrm{cone}\,}}(\{r\}),$ for all $x,y \in C$.

The *Minkowski sum* of two sets *X* and *Y* is defined as $X + Y = \{x + y \mid x\in X, y\in Y\}$. The next result states that any polyhedral cone can be decomposed into a Minkowski sum of its lineality space and a pointed cone.

### Proposition 2.2

Let $C\subseteq \mathbb {R}^n$ be a polyhedral cone, $L={{\,\mathrm{lin.space}\,}}(C)$. Let $G^1, \dots , G^s, s \ge 0$, be the distinct minimal proper faces of *C* and $g^i \in G^i {\setminus } L$, for $i=1,\dots ,s$. Let $P = {{\,\textrm{cone}\,}}(\{g^1, \dots , g^s\})$. Then

(i) *P* is a pointed cone and its extreme rays are ${{\,\textrm{cone}\,}}(\{g^1\}), \dots , {{\,\textrm{cone}\,}}(\{g^s\})$,
(ii) $C = L + P = L + {{\,\textrm{cone}\,}}(\{g^1, \dots , g^s\}), \; L \cap P = \{0\}$ and if $L \cap \text {lin}(P) = \{0\}$ then $\dim (C) = \dim (L) + \dim (P)$.

### Proof

(i) By definition *P* is a finitely generated cone. Assume that *P* is not pointed. Then there exist $\lambda _i \ge 0$, $i=1,\dots ,s$, not all equal to zero, such that $0=\sum _{i=1}^s\lambda _i\,g^i$. Hence, there exists $j \in \{1,\dots ,s\}$ such that $-g^j\in P\subseteq C$ and so $g^j\in L$, contradicting our choice.

To see that $g^1,\dots ,g^s$ define extreme rays of *P*, assume without loss of generality that ${{\,\textrm{cone}\,}}(\{g^1\})$ is not an extreme ray of *P*. Then we can find $\mu _i\ge 0$, $2\le i\le s$, not all equal to zero, such that $g^1=\sum _{i=2}^s \mu _i\,g^i$. As $G^1$ is a face, there exists a supporting hyperplane $H_a:= \{x \in \mathbb {R}^n \mid ax= 0\},a\in \mathbb {R}^n{\setminus }\{0\}$, such that $G^1 = H_a \cap C$ and $ax>0$ for all $x\in C{\setminus } G^1$. Thus, from $0=ag^1 = \sum _{i=2}^s\mu _i ag^i$ we conclude that $ag^k=0$ for some $k\in \{2,\dots ,s\}$. Therefore $g^k\in G^1$ and $G^k\subseteq G^1$, which leads to $G^k=G^1$, because $G^1$ is a minimal proper face. But then $G^1, \dots , G^s$ are not distinct, which is a contradiction.

(ii) By Theorem 8.5 in (Schrijver 1986), we have $C = L + P$. Since *L* is a face of *C*, there exists $a\in \mathbb {R}^n{\setminus }\{0\}$ such that $ax=0$ for all $x\in L$ and $ax>0$ for all $x\in C{\setminus } L$. From $ag^i > 0, i = 1,\dots ,s$, we get $ax > 0$, for all $x \in P {\setminus } \{0\}$, hence $L\cap P=\{0\}$. If $L \cap \text {lin}(P) = \{0\}$, we have $\text {lin}(C) = L\oplus \text {lin}(P)$, where $L\oplus \text {lin}(P)$ is the direct sum of the vector spaces *L* and $\text {lin}(P)$, and so $\dim (C) = \dim (L) + \dim (P).$ $\square$

The combinatorial type of the pointed cone $P={{\,\textrm{cone}\,}}(\{g^1,\dots , g^s\})$ in Proposition 2.2 is (in general) not uniquely determined. However, if we choose $g^1,\dots ,g^s$ such that $L\cap \text {lin}(P)=\{0\}$, i.e., all the $g^i$ are contained in some linear subspace $L'$ complementary to *L*, then the combinatorial type of *P* is independent of the choice of the $g^i$ from the minimal proper faces. Observe that for any complementary space $L'$ of *L*, $L'\cap G^i$ is a ray, i.e., $L'\cap G^i={{\,\textrm{cone}\,}}(\{g^i\})$ for some $g^i\in L'\cap G^i$.

### Proposition 2.3

Let $C\subseteq \mathbb {R}^n$ be a polyhedral cone, $L={{\,\mathrm{lin.space}\,}}(C)$, and let $P_1$, $P_2$ be pointed cones with $L+P_1 = C = L+P_2$ and $L \cap \text {lin}(P_1) = \{0\} = L \cap \text {lin}(P_2)$. Then $P_1$ and $P_2$ are combinatorially equivalent.

### Proof

Without loos of generality let $\dim (C)=n$ and $\dim (L)=t$. With $L'_j:=\text {lin}(P_j)$, by Proposition. 2.2 we have $\dim (L'_j)=n-t$ and $C\cap L'_j=P_j$, for $j=1,2$. Let $u^1,\dots ,u^{n-t}$ be a basis of $L'_1$. As also $L'_2$ is a complement of *L*, there exist uniquely determined $v^1,\dots ,v^{n-t}\in L'_2$, $w^{1},\dots , w^{n-t}\in L$ such that $u^i=v^i+w^i$, $1\le i\le n-t$. Now let $T: \mathbb {R}^n \rightarrow \mathbb {R}^n$ be the invertible linear map with

$$\begin{aligned} T(u) = u, \hspace{5.0pt}\forall u \in L, \quad T(u^i) =v^i, \hspace{5.0pt}1 \le i \le n-t. \end{aligned}$$

Then we get $T(C) = C$. To see this let $y = u + w \in C$ with $u\in L'_1$ and $w\in L$. Then we may write

$$\begin{aligned} y = \sum _{i=1}^{n-t} \lambda _i u^i + w, \text { for some } \lambda _1,\dots , \lambda _{n-t} \in \mathbb {R}. \end{aligned}$$

Thus

$$\begin{aligned} T(y)= & {} \sum _{i = 1}^{n-t} \lambda _iv^i + w = \sum _{i = 1}^{n-t} \lambda _i (u^i - w^i) + \left( y - \sum _{i=1}^{n-t} \lambda _i u^i\right) \\= & {} y - \sum _{i=1}^{n-t} \lambda _i w^i \in C+L=C, \end{aligned}$$

and vice versa. We conclude

$$\begin{aligned} T(P_1) = T(L'_1 \cap C) = T(L'_1) \cap T(C) = L'_2 \cap C = P_2. \end{aligned}$$

Thus, $P_1$ and $P_2$ are affinely and thereby also combinatorially equivalent. $\square$

We also point out that the relative interior of a polyhedral cone can easily be described by looking at the implicit equalities in $Ax \ge 0$:

### Proposition 2.4

Let $C = \{x \in \mathbb {R}^n \mid Ax \ge 0\} = \{x \in \mathbb {R}^n \mid A^=x=0, A^+x \ge 0\}$ be a polyhedral cone. Then

$$\begin{aligned} \textrm{relint}(C)= \{x \in \mathbb {R}^n \mid A^=x=0, A^+x > 0\}. \end{aligned}$$

### Proof

If $x\in C$ with $A^+x>0$ then for any $y\in \text {lin}(C) = \{y \in \mathbb {R}^n \mid A^=y=0\}$ there exists $\epsilon >0$ such that $A^+(x+\epsilon y) > 0$. Hence $x\in \textrm{relint}(C)$. Conversely, let $x\in \textrm{relint}(C)$ and let *a* be an arbitrary row of $A^+$. By definition of $A^+$ there exists $z\in C$ with $az>0$. As $x\in \textrm{relint}(C)$, there exists $\epsilon >0$ such $x-\epsilon z \in C$ and so $a(x-\epsilon z)\ge 0$. Thus $ax>0$. $\square$

## Metabolic networks

**Fig. 1:**
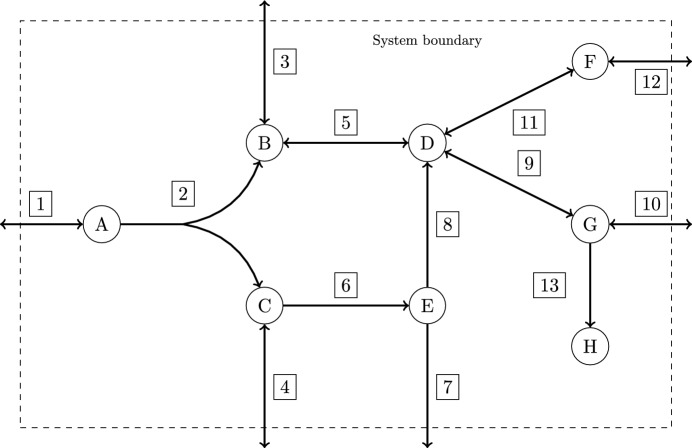
Example of a metabolic network

A *metabolic network* $\mathcal {N} = (\mathcal {M}, \mathcal {R}, S, {{\,\textrm{Irr}\,}})$ is given by a set $\mathcal {M}$ of (internal) *metabolites*, a set $\mathcal {R}= {{\,\textrm{Rev}\,}}\cup {{\,\textrm{Irr}\,}}$ of *reversible* and *irreversible reactions*, and a *stoichiometric matrix* $S \in \mathbb {R}^{m \times n}$, where $m = |\mathcal {M}|$ and $n = |\mathcal {R}|$. For $J \subseteq \mathcal {R}$, we denote by $S_{\star ,J}$ the submatrix of *S* whose columns belong to *J*.

The network can be seen as a weighted hypergraph with the generalized incidence matrix *S*, where the metabolites are represented by nodes and the reactions by hyperarcs. A positive entry $S_{i,j} > 0$ in the stoichiometric matrix *S* indicates that reaction *j* produces metabolite *i*. If $S_{i,j} < 0$, metabolite *i* is consumed in reaction *j*.

### Example 3.1

The metabolic network in Fig. 1 has the set of metabolites $\mathcal {M}= \{\mathrm {A,B,\dots ,G,H}\}$, the set of reversible reactions ${{\,\textrm{Rev}\,}}= \{1,3,4,5,9,10,11,12\}$ and the set of irreversible reactions ${{\,\textrm{Irr}\,}}= \{2,6,7,8,13\}$. The stoichiometric matrix is

$$\begin{aligned} S = \left( \begin{array}{rrrrrrrrrrrrr} 1 &{} -1 &{} 0 &{} 0 &{} 0 &{} 0 &{} 0 &{} 0 &{} 0 &{} 0 &{} 0 &{} 0 &{} 0 \\ 0 &{} 1 &{} \hspace{5.0pt}1 &{} 0 &{} -1 &{} 0 &{} 0 &{} 0 &{} 0 &{} 0 &{} 0 &{} 0 &{} 0\\ 0 &{} 1 &{} 0 &{} -1 &{} 0 &{} -1 &{} 0 &{} 0 &{} 0 &{} 0 &{} 0 &{} 0 &{} 0 \\ 0 &{} 0 &{} 0 &{} 0 &{} 1 &{} 0 &{} 0 &{} 1 &{} -1 &{} 0 &{} -1 &{} 0 &{} 0\\ 0 &{} 0 &{} 0 &{} 0 &{} 0 &{} 1 &{} -1 &{} -1 &{} 0 &{} 0 &{} 0 &{} 0 &{} 0\\ 0 &{} 0 &{} 0 &{} 0 &{} 0 &{} 0 &{} 0 &{} 0 &{} 0 &{} 0 &{} 1 &{} -1 &{} 0\\ 0 &{} 0 &{} 0 &{} 0 &{} 0 &{} 0 &{} 0 &{} 0 &{} 1 &{} -1 &{} 0 &{} 0 &{} -1 \\ 0 &{} 0 &{} 0 &{} 0 &{} 0 &{} 0 &{} 0 &{} 0 &{} 0 &{} 0 &{} 0 &{} 0 &{} 1 \end{array} \right) , \end{aligned}$$

where we assume that reversible reactions are oriented from left to right and from top to bottom. Furthermore, for simplicity, all stoichiometric coefficients are supposed to be 0,1, or $-1$.

### Flux cones

In metabolic network analysis, we often assume that the network is in *steady-state*, i.e., for each internal metabolite the rate of production is equal to the rate of consumption. In matrix notation, the steady-state constraints can be written as $Sv=0$, where $v \in \mathbb {R}^n$ denotes a *flux vector*. By adding the *thermodynamic irreversibility* constraints $v_j \ge 0$, for all $j \in {{\,\textrm{Irr}\,}},$ and setting

1 $$\begin{aligned} A = \begin{pmatrix} ~S~\\ -S~\\ I_{{{\,\textrm{Irr}\,}},\star } \end{pmatrix} \end{aligned}$$

we obtain the polyhedral cone

2 $$\begin{aligned} C = \{x \in \mathbb {R}^n \mid Ax \ge 0\} = \{v \in \mathbb {R}^n \mid Sv = 0,v_{{{\,\textrm{Irr}\,}}} \ge 0\}, \end{aligned}$$

which is called the (steady-state) *flux cone* of $\mathcal {N}$. Here, $v_{{{\,\textrm{Irr}\,}}}$ is the subvector of *v* whose components belong to ${{\,\textrm{Irr}\,}}$, and $I_{{{\,\textrm{Irr}\,}},\star }$ the submatrix of the $(n \times n)$ identity matrix $I_n$ whose rows correspond to the irreversible reactions.

*Implicit equalities* The implicit equalities in the definition (2) of a flux cone *C* include all steady-state constraints $Sv=0$. If any of the irreversibility constraints $v_j \ge 0, j \in {{\,\textrm{Irr}\,}}$, is an implicit equality, the corresponding reaction $j \in {{\,\textrm{Irr}\,}}$ is *blocked*, i.e., $v_j = 0$, for all $v \in C$. For some of our results, we will assume that there are no implicit equalities in $v_{{{\,\textrm{Irr}\,}}} \ge 0$, or equivalently that there are no blocked irreversible reactions. Blocked reactions can be determined by solving a linear optimization problem.

*Redundant inequalities* If in (2) one of the irreversibility constraints $v_j \ge 0, j \in {{\,\textrm{Irr}\,}},$ is redundant, the corresponding reaction *j* can be shifted from the set ${{\,\textrm{Irr}\,}}$ of irreversible reactions to the set ${{\,\textrm{Rev}\,}}$ of reversible reactions, without changing the flux cone *C*. The constraint $v_j \ge 0$ is then implied by the remaining constraints.

#### Example 3.2

In the metabolic network of Fig. 1, the irreversible reaction 13 is blocked, i.e., $v_{13}=0$, for all $v \in C$, because there is no reaction to consume metabolite $\textrm{H}$.

The irreversibility constraint $v_6 \ge 0$ is redundant. There can be no flux from metabolite $\textrm{E}$ to metabolite $\textrm{C}$ because there is no reaction producing $\textrm{E}$.

The reversible reaction 1 cannot carry flux from right to left. Reaction 1 could be added to the set ${{\,\textrm{Irr}\,}}$ of irreversible reactions, but then the inequality $v_1 \ge 0$ would be redundant.

If redundant inequalities are removed from the description of a flux cone, the resulting irredundant description is in general not unique, because it depends on the order in which the redundant constraints are removed.

#### Proposition 3.3

Let $C = \{v \in \mathbb {R}^n \mid S v=0, v_{{{\,\textrm{Irr}\,}}} \ge 0 \}$ be a flux cone such that none of the inequalities $v_j \ge 0, j \in {{\,\textrm{Irr}\,}},$ is redundant or an implicit equality. Then *C* has exactly $|{{{\,\textrm{Irr}\,}}} |$ facets and each facet *F* has the representation

3 $$\begin{aligned} F = \{v \in C \mid v_j = 0\}, \text { for some j} \in {{\,\textrm{Irr}\,}}. \end{aligned}$$

#### Proof

If there are no implicit equalities in $v_{{{\,\textrm{Irr}\,}}} \ge 0$ and *A* is given by (1), then $$A^= = \begin{pmatrix} ~S~\\ -S~ \end{pmatrix}$$ and $A^+ = I_{{{\,\textrm{Irr}\,}},\star }$. Since there are no redundant inequalities in $v_{{{\,\textrm{Irr}\,}}} \ge 0$, the result follows from Theorem 8.1 in Schrijver (1986). $\square$

### Elementary flux modes

In metabolic network analysis we are particularly interested in minimal functional units of the network. Let *C* be the flux cone of a metabolic network $\mathcal {N}$. A vector $e \in C {\setminus } \{0\}$ is called an *elementary flux mode* (EFM) (Schuster and Hilgetag 1994) if it has inclusionwise minimal support, i.e., if

4 $$\begin{aligned} \forall v \in C \setminus \{0\}: {{\,\textrm{supp}\,}}(v) \subseteq {{\,\textrm{supp}\,}}(e) \implies {{\,\textrm{supp}\,}}(v) = {{\,\textrm{supp}\,}}(e), \end{aligned}$$

where the *support* of $v \in \mathbb {R}^n$ is defined by ${{\,\textrm{supp}\,}}(v) = \{ i \in \mathcal {R}\mid v_i \ne 0\}.$ We say that a reaction $i \in \mathcal {R}$ is *active* in $v \in C$ if $i \in {{\,\textrm{supp}\,}}(v)$. By ${{\,\mathrm{irr.supp}\,}}(v):= {{\,\textrm{supp}\,}}(v) \cap {{\,\textrm{Irr}\,}}$ we denote the *irreversible support* of *v*, i.e., the set of active irreversible reactions in *v*. Analogously, ${{\,\mathrm{rev.supp}\,}}(v):= {{\,\textrm{supp}\,}}(v) \cap {{\,\textrm{Rev}\,}}$ denotes the *reversible support* of *v*.

To verify whether a given flux vector $v \in C$ is an EFM the *rank test* (Jevremovic et al. 2010; Urbanczik and Wagner 2005) can be applied, i.e.,

5 $$\begin{aligned} v \in C \text { is an EFM if and only if } {{\,\textrm{rank}\,}}(S_{\star ,{{\,\textrm{supp}\,}}(v)}) = |{{\,\textrm{supp}\,}}(v) |-1. \end{aligned}$$

#### Proposition 3.4

Let $C = \{v \in \mathbb {R}^n \mid Sv = 0, v_{{{\,\textrm{Irr}\,}}} \ge 0 \}$ be the flux cone of a metabolic network. Then

$$\begin{aligned} |{{\,\mathrm{irr.supp}\,}}(e) |\le {{\,\textrm{rank}\,}}(S_{\star ,{{\,\textrm{Irr}\,}}}) + 1, \end{aligned}$$

for each EFM $e \in C$.

#### Proof

Suppose the opposite. Then

$$\begin{aligned} {\begin{matrix} |{{\,\textrm{supp}\,}}(e) |&{} = |{{\,\mathrm{irr.supp}\,}}(e) |+ |{{\,\mathrm{rev.supp}\,}}(e) |\\ &{}\ge {{\,\textrm{rank}\,}}(S_{\star ,{{\,\textrm{Irr}\,}}}) + 2+ |{{\,\mathrm{rev.supp}\,}}(e) |\\ &{}\ge {{\,\textrm{rank}\,}}(S_{\star ,{{\,\mathrm{irr.supp}\,}}(e)}) +2 + {{\,\textrm{rank}\,}}(S_{\star ,{{\,\mathrm{rev.supp}\,}}(e)}) \\ &{}\ge {{\,\textrm{rank}\,}}(S_{\star ,{{\,\textrm{supp}\,}}(e)}) +2, \end{matrix}} \end{aligned}$$

contradicting the rank test (5). $\square$

**Table 1:** Characteristics of the three example networks

	E. coli core	Pentose phosphate	Pyruvate
$(m,n)= (|\mathcal {M}|,|\mathcal {R}|)$	(72, 94)	(34, 57)	(28, 81)
$|{{\,\textrm{Irr}\,}}|$	48	19	40
$|{{\,\textrm{Rev}\,}}|$	46	38	41
${{\,\textrm{rank}\,}}(S)$	67	34	28
$n-{{\,\textrm{rank}\,}}(S)$	27	23	53
$\dim (C)$	23	23	53
$t=\dim (L)$	0	8	16
$\dim (P)$	23	15	37
$|\text {Facets} |$	39	17	37
$|\text {blocked irr} |$	8	0	0
$|\text {blocked rev}|$	2	0	0
$|\text {EFMs} |$	16673	5180	47854
$|\text {MMBs} |$	1421	19	37

$|\mathcal {M}|$ and $|\mathcal {R}|$ denote the number of metabolites resp. reactions, which correspond to the number of rows resp. columns of the stoichiometric matrix. $|{{\,\textrm{Irr}\,}}|$ and $|{{\,\textrm{Rev}\,}}|$ denote the number of irreversible resp. reversible reactions of the network. ${{\,\textrm{rank}\,}}(S)$ is the rank of the stoichiometric matrix. The flux cone $C = L + P$ is the Minkowski sum of the lineality space *L* and a pointed cone *P*, with $\dim (C) = \dim (L) + \dim (P)$. $|\text {Facets} |$ is the number of facets of the flux cone, which is equal to the number of irreversibility constraints if none of these is redundant or an implicit equality. $|\text {blocked irr} |$ resp. $|\text {blocked rev} |$ describe the number of blocked irreversible resp. blocked reversible reactions. $|\text {EFMs} |$ is the number of EFMs and $|\text {MMBs} |$ the number of minimal metabolic behaviors (cf. Sect. 7)

### Illustrative examples

To illustrate the theoretical results in the following sections through concrete examples, we will use the metabolic networks Pyruvate and Pentose Phosphate Pathway from the KEGG database (https://www.genome.jp/kegg/pathway.html, (Kanehisa and Goto 2000)) and Escherichia coli str. K-12 substr. MG1655 (E.coli core) from the BiGG database (King et al. 2016), where we removed the biomass reaction. The characteristics of these networks are summarized in Table 1. The EFMs were computed with efmtool (https://csb.ethz.ch/tools/software/efmtool.html, (Terzer 2009)). For polyhedral computations we used polymake (https://polymake.org/, (Assarf et al. 2017)).

## Faces of the flux cone and metabolic behaviors

Given a metabolic network $\mathcal {N}$ with flux cone *C*, a *metabolic behavior* (Larhlimi and Bockmayr 2009) of *C* is a nonempty set of irreversible reactions $D \subseteq {{\,\textrm{Irr}\,}}$ with $D = {{\,\mathrm{irr.supp}\,}}(v)$, for some $v \in C$. A *minimal metabolic behavior* (MMB) is a metabolic behavior *D* for which there is no other metabolic behavior $D' \subsetneq D$. Larhlimi and Bockmayr (2009) have shown that minimal metabolic behaviors are in a 1–1 correspondence with the minimal proper faces of the flux cone *C*. In particular, if *G* is a minimal proper face and *L* the lineality space of *C*, then all flux vectors $v \in G \setminus L$ have the same irreversible support $D_G = {{\,\mathrm{irr.supp}\,}}(v)$, which is a minimal metabolic behavior.

### Proposition 4.1

Let *C* be the flux cone of a metabolic network. Then each metabolic behavior is the union of MMBs.

### Proof

Let $\emptyset \ne D \subseteq {{\,\textrm{Irr}\,}}$ be a metabolic behavior and let $v \in C$ with $D={{\,\mathrm{irr.supp}\,}}(v)$. Let *L* be the lineality space and $G^1,\dots ,G^s, s \ge 0,$ be the minimal proper faces of *C*. Since $D \ne \emptyset$ and ${{\,\mathrm{irr.supp}\,}}(l) = \emptyset$, for all $l \in L$, we have $C \ne L$. Thus *C* has at least one minimal proper face and $s \ge 1$. By Proposition 2.2, $v = l + \sum _{i=1}^s \lambda _i g^i$, for some $l \in L$ and $\lambda _i \ge 0, g^i \in G^i {\setminus } L$, for $i = 1,\dots ,s$. It follows ${{\,\mathrm{irr.supp}\,}}(v) = \bigcup _{\lambda _i> 0} {{\,\mathrm{irr.supp}\,}}(g^i) = \bigcup _{\lambda _i > 0} D^i$, where $D^i = {{\,\mathrm{irr.supp}\,}}(g^i)$ is the MMB of the minimal proper face $G^i$, for $i=1,\dots ,s$. $\square$

Next we generalize the characterization of minimal proper faces by MMBs (Larhlimi and Bockmayr 2009) to higher-dimensional faces.

### Proposition 4.2

Let *C* be the flux cone of a metabolic network and let *F* be a face of *C*. Then all $v \in {{\,\textrm{relint}\,}}(F)$ have the same irreversible support or equivalently share the same metabolic behavior.

### Proof

Let $v,w\in {{\,\textrm{relint}\,}}(F)$. Assume w.l.o.g. that there exists $j \in {{\,\mathrm{irr.supp}\,}}(w) {\setminus } {{\,\mathrm{irr.supp}\,}}(v)$. Then $v_j=0$, but $w_j>0$, and hence for any $\lambda >1$, we have $\lambda v+(1-\lambda )w\notin C$. However, since $v,w\in {{\,\textrm{relint}\,}}(F)$, we know $\lambda v+(1-\lambda )w\in F$, for some $\lambda >1$. This shows ${{\,\mathrm{irr.supp}\,}}(v)={{\,\mathrm{irr.supp}\,}}(w)$, which implies the statement.$\square$

### Example 4.3

**Fig. 2:**
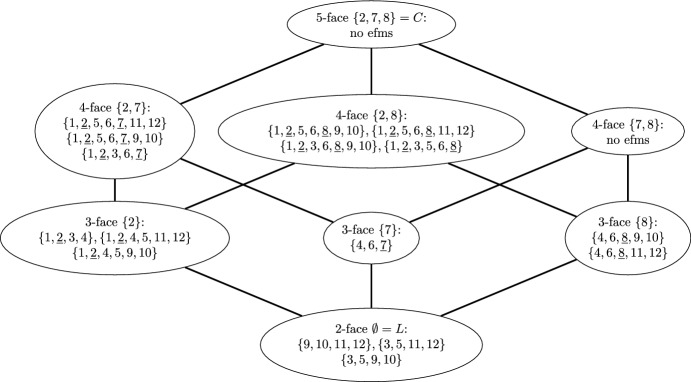
Face lattice of the network in Fig. 1. Each node represents a face of the flux cone together with the corresponding metabolic behavior and the EFMs contained in that face. The active irreversible reactions are underlined. Edges connecting the nodes indicate the inclusion of lower-dimensional in higher-dimensional faces. The only 2-face is the lineality space *L* and the only 5-face is the entire flux cone *C*

Consider the network in Fig. 1. If we remove the redundant irreversibility constraint $v_6 \ge 0$ and assume $6 \in {{\,\textrm{Rev}\,}}$, the MMBs of the network are $\{2\},\{7\},\{8\}$ (if $6 \in {{\,\textrm{Irr}\,}}$, the MMBs are $\{2\},\{6,7\},\{6,8\}$). The face lattice together with the EFMs contained in each face is shown in Fig. 2.

## The degree of flux vectors

Let *C* be the flux cone of a metabolic network. We define the *degree* $\deg (v)$ of a flux vector $v \in C$ as the dimension of the inclusionwise minimal face of *C* containing *v*, which for $v \ne 0$ is the unique face *F* of *C* with $v \in {{\,\textrm{relint}\,}}(F)$. By Proposition 2.1, we have

6 $$\begin{aligned} \deg (v) = n - {{\,\textrm{rank}\,}}(A_v^=), \quad \text { where } \quad A^=_v = \begin{pmatrix} S \\ -S \\ I_{{{\,\textrm{Irr}\,}}\setminus {{\,\mathrm{irr.supp}\,}}(v),\star } \end{pmatrix}. \end{aligned}$$

It follows that flux vectors in the lineality space *L* of *C* have degree $\dim (L)$ and flux vectors in minimal proper faces have degree $\dim (L)+1$. A flux vector in the relative interior of *C* has degree $\dim (C)$.

A widely used technique in metabolic network analysis is to split reversible reactions into two irreversible reactions (Clarke 1980; Schilling et al. 2000; Papin et al. 2004; Gagneur and Klamt 2004; Röhl and Bockmayr 2019). In particular, this is applied in algorithms for EFM computation such as efmtool (Terzer 2009) or EFMlrs (Buchner and Zanghellini 2021). In these algorithms, splitting all reversible reactions leads to a pointed cone $C'$ in a higher-dimensional space. The EFMs of the original flux cone *C* correspond to extreme rays in the reconfigured cone $C'$ (Gagneur and Klamt 2004), which all have degree 1 in $C'$. Therefore, the degree of an EFM as defined by (6) has to be determined in the original flux cone *C* and not in $C'$.

Next we further characterize flux vectors in the relative interior of *C*.

### Proposition 5.1

Let $C = \{v \in \mathbb {R}^n \mid Sv = 0, v_{{{\,\textrm{Irr}\,}}} \ge 0\}$ be a flux cone with no implicit equalities in $v_{{{\,\textrm{Irr}\,}}} \ge 0$. For $v \in C$ we have $\deg (v)=\dim (C)$ if and only if $v_i > 0$ for all $i \in {{\,\textrm{Irr}\,}}$.

### Proof

Direct consequence of Proposition 2.4. $\square$

Although small examples with EFMs in the relative interior of the flux cone can be constructed, we note that real biological networks typically do not have EFMs where all unblocked irreversible reactions are active. In these cases, there are no EFMs in the relative interior of the cone, i.e., all EFMs lie on the relative boundary of *C*.

Next we prove an upper bound on the degree of flux vectors.

### Proposition 5.2

Let $C = \{ v \in \mathbb {R}^n \mid Sv = 0, v_{{{\,\textrm{Irr}\,}}} \ge 0 \}$ be the flux cone of a metabolic network with lineality space *L*. Then for each flux vector $v \in C$

$$\begin{aligned} \deg (v) \; \le \; \dim (L) + |{{\,\mathrm{irr.supp}\,}}(v) |. \end{aligned}$$

### Proof

By definition of the lineality space *L*, $t:= \dim (L) = n - {{\,\textrm{rank}\,}}(A)$, with $$A = \begin{pmatrix} S \\ -S \\ I_{{{\,\textrm{Irr}\,}},\star } \end{pmatrix}$$.

By (6), we have $\deg (v) = n - {{\,\textrm{rank}\,}}(A^=_v)$, with $$A^=_v = \begin{pmatrix} S \\ -S \\ I_{{{\,\textrm{Irr}\,}}\setminus {{\,\mathrm{irr.supp}\,}}(v),\star } \end{pmatrix}$$.

It follows ${{\,\textrm{rank}\,}}(A) - {{\,\textrm{rank}\,}}(A^=_v) = (n-t) - (n - \deg (v)) = \deg (v)-t$. Thus, at least $\deg (v)-t$ rows from *A* must be missing in $A_v^=$. This implies $|{{\,\mathrm{irr.supp}\,}}(v) |\ge \deg (v)-t$ or $\deg (v) \le t + |{{\,\mathrm{irr.supp}\,}}(v) |$. $\square$

By combining Proposition 3.4 and Proposition 5.2, we get an upper bound on the degree of EFMs.

### Corollary 5.3

Let $C = \{ v \in \mathbb {R}^n \mid Sv = 0, v_{{{\,\textrm{Irr}\,}}} \ge 0 \}$ be the flux cone of a metabolic network with lineality space *L*. Then for each EFM $e \in C$

$$\begin{aligned} \deg (e) \; \le \; \dim (L) + ({{\,\textrm{rank}\,}}(S_{\star ,{{\,\textrm{Irr}\,}}})+1). \end{aligned}$$

The following example shows that the bound from Corollary 5.3 is sharp.

**Fig. 3:**
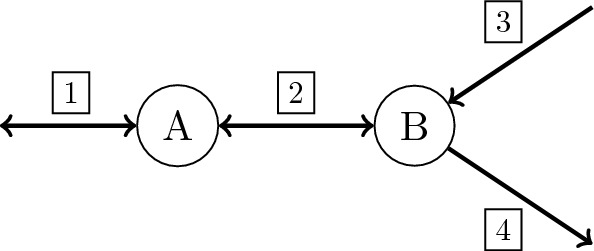
Example network

**Table 2:** Maximum number of active irreversible reactions and maximum degree of EFMs together with the upper bounds from Proposition 3.4, 5.2 and Corollary 5.3

	E. coli core	Pentose phosphate	Pyruvate
($|\mathcal {M}|,|\mathcal {R}|$)	(72, 94)	(34, 57)	(28, 81)
$|{{\,\textrm{Irr}\,}}|$	48	19	40
$|{{\,\textrm{Rev}\,}}|$	46	38	41
${{\,\textrm{rank}\,}}(S)$	67	34	28
${{\,\textrm{rank}\,}}(S_{\star ,{{\,\textrm{Irr}\,}}})$	41	16	24
$t=\dim ({{\,\mathrm{lin.space}\,}}(C))$	0	8	16
$q=\max \{|{{\,\mathrm{irr.supp}\,}}(e) |:e \text { EFM}\}$	23	9	10
$r = {{\,\textrm{rank}\,}}(S_{\star ,{{\,\textrm{Irr}\,}}})+1$ (cf. Proposition 3.4)	42	17	25
$\max \{\deg (e): e \text { EFM}\}$	6	14	24
$t+q$ (cf. Proposition 5.2)	23	17	26
$t+r$ (cf. Corollary 5.3)	42	25	41
$\dim (C)$	23	23	53
$|\text {EFMs} |$	16673	5180	47854

### Example 5.4

The network in Fig. 3 contains 2 metabolites and 4 reactions, with ${{\,\textrm{Rev}\,}}= \{1,2\}$ and ${{\,\textrm{Irr}\,}}= \{3,4\}$. Given the stoichiometric matrix

$$\begin{aligned} S =\begin{pmatrix} 1&{}-1&{}0&{}0\\ 0&{}1&{}1&{}-1 \end{pmatrix} \end{aligned}$$

the network has the EFMs $e^1=(1,1,0,1), \; e^2=(-1,-1,1,0)$ and $e^3 = (0,0,1,1)$, with $\deg (e^1) = \deg (e^2) = 1$ and $\deg (e^3) = 2$. Note that $C = {{\,\textrm{cone}\,}}(\{e^1, e^2\})$ and $e^3 = e^1 + e^2 \in {{\,\textrm{relint}\,}}(C)$. Since there are no reversible flux vectors, we have $\dim ({{\,\mathrm{lin.space}\,}}(C)) = 0$. Furthermore, ${{\,\textrm{rank}\,}}(S_{\star ,{{\,\textrm{Irr}\,}}}) = 1$ and thus $\deg (e^3) = \dim ({{\,\mathrm{lin.space}\,}}(C)) + ({{\,\textrm{rank}\,}}(S_{\star ,{{\,\textrm{Irr}\,}}})+1) = 2$.

**Fig. 4:**
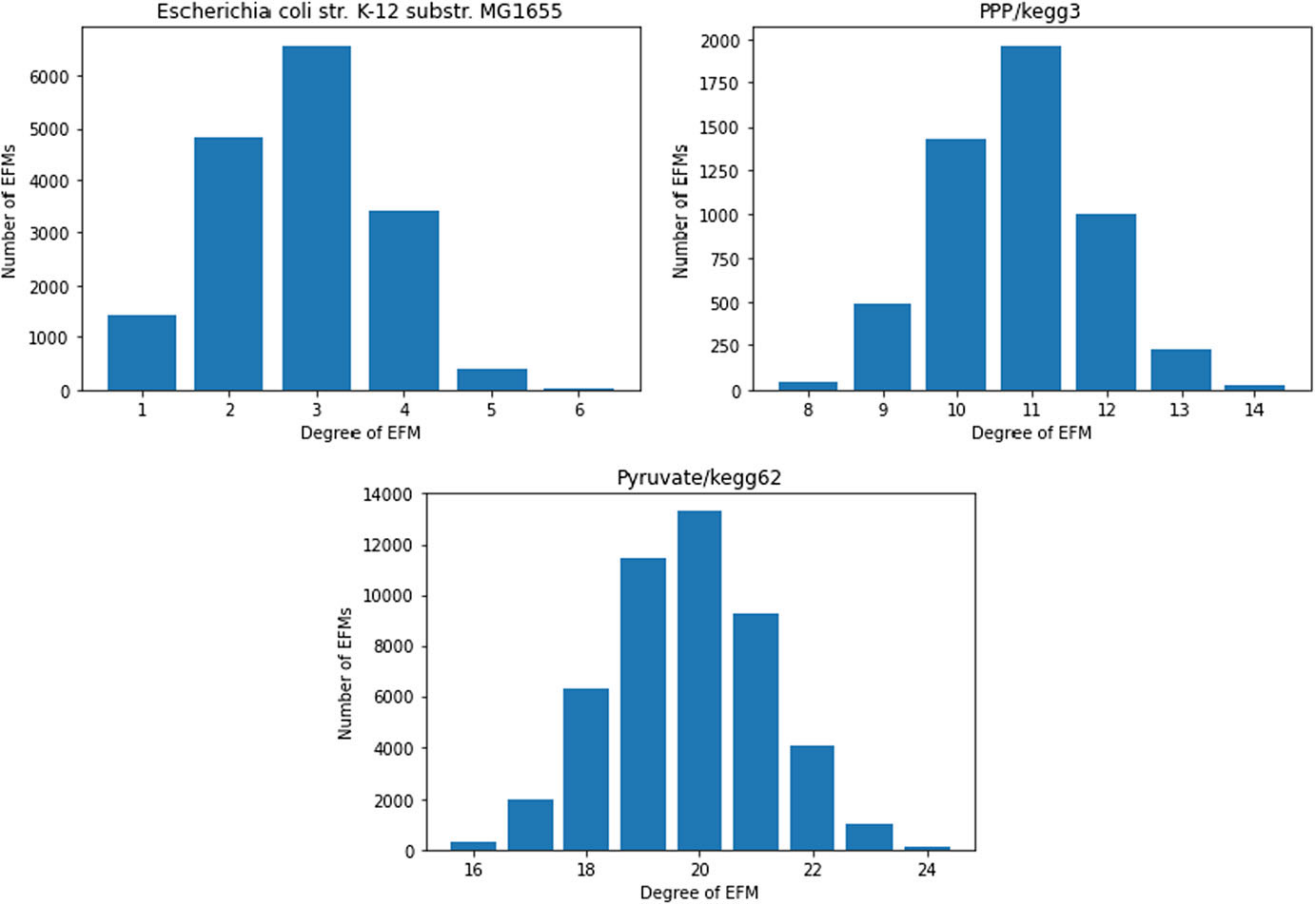
Degree distribution of EFMs

For the example networks E.coli core, Pentose Phosphate Pathway and Pyruvate from Sect. 3.3, the maximum degree of EFMs and the upper bounds from Proposition 5.2 and Corollary 5.3 are given in Table 2. We can see that $l=\max \{ |{{\,\mathrm{irr.supp}\,}}(e) |:e \text { EFM}\}$ is much smaller than the upper bound ${{\,\textrm{rank}\,}}(S_{\star ,{{\,\textrm{Irr}\,}}})+1$ from Proposition 3.4. The actual degrees of the EFMs in these networks are summarized in Fig. 4.

The next proposition explains the scarcity of EFMs in the relative interior of a flux cone *C*, in the facets of *C* and in the faces of dimension $\dim (C)-2$.

### Proposition 5.5

Let $C = \{v \in \mathbb {R}^n \mid Sv = 0, v_{{{\,\textrm{Irr}\,}}} \ge 0\}$ be a flux cone with no redundant inequalities or implicit equalities in $v_{{{\,\textrm{Irr}\,}}} \ge 0$. If $|{{\,\textrm{Irr}\,}}|> {{\,\textrm{rank}\,}}(S_{\star ,{{\,\textrm{Irr}\,}}})+q$, for some $q\in \{1,2,3\}$, then $\deg (e)\le \dim (C)-q$, for each EFM *e* of *C*.

### Proof

By Prop. 3.4, $|{{\,\mathrm{irr.supp}\,}}(e) |\le {{\,\textrm{rank}\,}}(S_{\star ,{{\,\textrm{Irr}\,}}})+1$, for each EFM *e* of *C*.

Assume $\deg (e) = \dim (C)-(q-1)$, for some $q \in \{1,2,3\}$. Then, by definition, the inclusionwise minimal face *F* containing *e* has dimension $\dim (C) - (q-1) \ge \dim (C) - 2$. It follows that *e* resp. *F* is contained in exactly $q-1$ facets of *C*. Here we use for $q=3$ that a $(\dim (C)-2)$-face is contained in exactly two facets, cf. (Schrijver 1986, p.105).

By the hypothesis on the description of the cone, it follows $|{{\,\mathrm{irr.supp}\,}}(e) |= |{{\,\textrm{Irr}\,}}|- (q-1)$. So we get $|{{\,\textrm{Irr}\,}}|- (q-1) \le {{\,\textrm{rank}\,}}(S_{\star ,{{\,\textrm{Irr}\,}}})+1$ or $|{{\,\textrm{Irr}\,}}|\le {{\,\textrm{rank}\,}}(S_{\star ,{{\,\textrm{Irr}\,}}})+q$, in contradiction to the hypothesis $|{{\,\textrm{Irr}\,}}|> {{\,\textrm{rank}\,}}(S_{\star ,{{\,\textrm{Irr}\,}}})+q.$ $\square$

In the proof we used that $(\dim (C)-q)$-faces of a cone *C* are contained in exactly *q* facets of *C*, for $q = 0,1,2$. As the example of a 3-dimensional pointed cone with *n* facets shows, a $(\dim (C) - 3)$-face (here the origin) can be contained in an arbitrary number of facets, and thus a similar argument does not hold for such faces. To limit the number of facets a face can be contained in, we introduce the concept of *l-simple* cones and use this for a generalization of Prop. 5.5.

A cone $C\subseteq \mathbb {R}^n$ is called *l*-*simple* for some $l \ge 1$, if every *k*-face of *C* is contained in at most $l \cdot (\dim (C) - k)$ facets of *C*, for all $k = \dim ({{\,\mathrm{lin.space}\,}}(C)), \dots , \dim (C)$. Assuming that a flux cone is *l*-simple leads to another bound on the degree of EFMs.

### Proposition 5.6

Let $C = \{v \in \mathbb {R}^n \mid Sv = 0, v_{{{\,\textrm{Irr}\,}}} \ge 0 \}$ be an *l*-simple cone with no redundant inequalities or implicit equalities in $v_{{{\,\textrm{Irr}\,}}} \ge 0$. Then for each EFM $e \in C$

$$\begin{aligned} \deg (e) \le \dim (C) - \frac{|{{\,\textrm{Irr}\,}}|-({{\,\textrm{rank}\,}}(S_{\star ,{{\,\textrm{Irr}\,}}}) +1)}{l}. \end{aligned}$$

### Proof

By Prop. 3.4, $|{{\,\mathrm{irr.supp}\,}}(e) |\le {{\,\textrm{rank}\,}}(S_{\star ,{{\,\textrm{Irr}\,}}}) +1$ for each EFM $e \in C$ and thus at least $|{{\,\textrm{Irr}\,}}|- ({{\,\textrm{rank}\,}}(S_{\star ,{{\,\textrm{Irr}\,}}}) +1)$ entries of $v_{{{\,\textrm{Irr}\,}}}$ are equal to zero. Hence *e* is contained in at least $|{{\,\textrm{Irr}\,}}|- ({{\,\textrm{rank}\,}}(S_{\star ,{{\,\textrm{Irr}\,}}}) +1)$ facets of *C*. Suppose $\deg (e) = k$ and let $e \in F$, where *F* is a *k*-face of *C*. Since *C* is *l*-simple, *F* is contained in at most $l\cdot (\dim (C) - k)$ facets. It follows

$$\begin{aligned} |{{\,\textrm{Irr}\,}}|- ({{\,\textrm{rank}\,}}(S_{\star ,{{\,\textrm{Irr}\,}}}) + 1) \le l \cdot (\dim (C) - k) \end{aligned}$$

or

$$\begin{aligned} \deg (e) = k \; \le \; \dim (C) - \frac{|{{\,\textrm{Irr}\,}}|- ({{\,\textrm{rank}\,}}(S_{\star ,{{\,\textrm{Irr}\,}}}) + 1)}{l}. \end{aligned}$$

$$\square $$

Note that this bound is mainly theoretical because for the computation of *l* all faces of the flux cone have to be considered. Nevertheless $|{{\,\textrm{Irr}\,}}|\ge {{\,\textrm{rank}\,}}(S_{\star ,{{\,\textrm{Irr}\,}}})$ and $|{{\,\textrm{Irr}\,}}|$ is typically significantly larger than ${{\,\textrm{rank}\,}}(S_{\star ,{{\,\textrm{Irr}\,}}})$ (cf. Table 2).

## Decomposing flux vectors

Elementary flux modes have been introduced as minimal functional units of a metabolic network (Schuster and Hilgetag 1994; Schuster et al. 2000). Any vector $v \in C$ in the flux cone *C* of a metabolic network can be decomposed into a conic combination

7 $$\begin{aligned} v = \sum _{i=1}^m \lambda _i e^i, \text { with } \lambda _i > 0, \end{aligned}$$

of elementary flux modes $e^1,\dots ,e^m \in C$, see e.g. Lemma 1 in (Schuster et al. 2002). Note that in most cases, this decomposition is not unique.

Decomposing flux vectors into elementary flux modes has been widely used in metabolic pathway analysis, see e.g. (Poolman et al. 2004; Schwartz and Kanehisa 2005; Chan and Ji 2011; Jungers et al. 2011; Rügen et al. 2012; Kelk et al. 2012; Maarleveld et al. 2015; Oddsdóttir et al. 2015).

Here we show that the size and complexity of such a decomposition crucially depends on the degree of *v*. More precisely, we prove in Prop. 6.2 that any flux vector of degree *k* is a conic combination of at most *k* EFMs of degree at most *k*.

We first note that decomposing a flux vector belonging to some face $F \subseteq C$ is only possible by using vectors from the same face *F*.

### Proposition 6.1

Let $C = \{v \in \mathbb {R}^n \mid Sv = 0, v_{{{\,\textrm{Irr}\,}}} \ge 0 \}$ be the flux cone of a metabolic network. Let $F = \{v \in C \mid v_I = 0\}$, for some $I \subseteq {{\,\textrm{Irr}\,}}$, be a face of *C*.

If $v = \sum _{i=1}^m \lambda _i v^i, \lambda _i > 0,$ is a conic decomposition of a flux vector $v \in F$ into flux vectors $v^1, \dots , v^m \in C$, then $v^1, \dots , v^m \in F$.

### Proof

Suppose $v^{l} \not \in F,$ for some $l \in \{1,\dots ,m\}$. Then $v^{l}_j > 0$, for some $j \in I$. It follows $v_j = \sum _{i=1}^m \lambda _i v^i_j \ge \lambda _l v^{l}_j > 0$, and therefore $v \not \in F$. $\square$

Next we show that any flux vector $v \in C$ of degree *k* can be decomposed into a conic combination of at most *k* EFMs of degree at most $t+1$, where *t* is the dimension of the lineality space *L* of *C*. In other words, this means that any flux vector of degree *k* can be written as a non-negative sum of at most *k* EFMs, all belonging to the lineality space or to some minimal proper face of the flux cone. From each minimal proper face, at most one EFM is needed for the decomposition and all EFMs belonging to the same minimal proper face share the same minimal metabolic behavior (MMB). In the special case $k=t$, the EFMs in the decomposition all have degree *t* and belong to the lineality space (cf. Prop. 6.1).

### Proposition 6.2

Let $C \subseteq \mathbb {R}^n$ be the flux cone of a metabolic network and let $v \in C$ be a flux vector with $\deg (v) = k$. Let $F \subseteq C, \dim (F) = k,$ be the inclusionwise minimal face containing *v*. Then

i) *v* can be decomposed into a conic combination $v = \sum _{i=1}^{m} \lambda _i e^i, \lambda _i > 0,$ of at most $m \le k$ EFMs $e^i \in F$, with $\deg (e^i) \le t+1$, for $i=1,\dots ,m$, where *t* is the dimension of the lineality space *L* of *C*. In the special case $k=t$ and $F=L$, we have $e^i \in L$ and $\deg (e^i) = t$, for $i=1,\dots ,m$.
ii) with probability 1, *v* does not allow for a conic decomposition into $m < k$ EFMs.

### Proof

i): Since the EFMs generate the flux cone *C* (Schuster et al. (2002), Lemma 1), there exists a conic decomposition $v = \sum _{i=1}^m \lambda _i e^i, \lambda _i > 0$, for some EFMs $e^1,\dots ,e^m \in C$. By Prop. 2.2 we can assume that $e^1,\dots ,e^m$ belong to the lineality space or to the minimal proper faces of *C*, i.e., $\deg (e^i) \le t+1$, for $i=1,\dots ,m$. Using Carathéodory’s theorem, see e.g. (Lauritzen 2013, Prop. 3.14), we can also assume that the EFMs $e^1,\dots ,e^m$ in the conic decomposition (7) are linearly independent. By Prop. 6.1, it follows that $e^1,\dots ,e^m \in F$. Since $\dim (F) = k$, there can be at most *k* linearly independent vectors in *F*, thus $m \le k$ and the result follows.

ii): Using $k-1$ EFMs one can generate at most a $(k-1)$-dimensional subset of the *k*-dimensional face *F*. Since there are only finitely many ways of choosing $k-1$ EFMs in the set of all EFMs, the set of flux vectors $v \in F$ that can be decomposed into a combination of $k-1$ EFMs is the finite union of sets of *k*-dimensional volume 0 and therefore itself a set of *k*-dimensional volume 0 in *F*. This implies the result.$\square$

Note that Prop. 6.2 applies also to EFMs in higher-dimensional faces, for which we get the following result:

### Corollary 6.3

Let $e \in C$ be an EFM with $\deg (e) = k \ge t+2$, where *t* is the dimension of the lineality space *L* of *C*. Then *e* can be decomposed into a conic combination $e = \sum _{i=1}^m \lambda _i e^i, \lambda _i > 0, 2 \le m \le k$, of at least 2 and at most *k* EFMs of degree strictly smaller than *k*.

Since every *k*-face $F \subseteq C$ is the conic hull of the EFMs contained in *F*, we get the following lower bound on the number of EFMs in *F*.

### Proposition 6.4

Let $F \subseteq C$ be a face of the flux cone *C* with $\dim (F) = k$. Then *F* contains at least *k* EFMs. In particular, the cone *C* itself contains at least $\dim (C)$ EFMs.

To conclude this section we illustrate the decomposition of flux vectors of different degree on a concrete example from flux balance analysis (Orth et al. 2010).

### Example 6.5

We consider again the E.coli core model, now with the biomass reaction included. Using COBRApy (Ebrahim et al. 2013), we perform a flux balance analysis (FBA), where we determine the optimal growth rate while glucose uptake *Ex_glc__D_e* is limited by a lower bound of $-10$ (the number is negative because the exchange reactions in this model are oriented outwards). The remaining reactions have an upper bound of 1000 and lower bounds of $-1000$ resp. 0 for the reversible resp. irreversible reactions. The optimal growth rate of 0.917 is attained by a flux vector of degree 1, which is an extreme ray of the pointed flux cone and thus also an EFM.

Adding the lower bound 8.39 for the ATP-maintenance reaction *ATPM* leads to the standard version of the model when downloaded from the BiGG database (King et al. 2016). Now the optimal growth rate is 0.874 and the flux vector achieving this has degree 2. There is a unique decomposition into the 2 EFMs of degree 1 that span the 2-face containing this optimal solution.

If we perform a single knock-out of the gene *b1761 (gdhA)*, which blocks the reaction *GLUDy*, the new optimal growth rate is 0.851. The degree of the flux vector achieving this optimal growth rate changes to 3 and the 3-face containing this optimal solution is spanned by 3 EFMs of degree 1. Therefore, there is only one decomposition of this optimal solution into 3 EFMs of degree 1.

After knocking out a second gene *b3236 (mdh)*, which blocks the reaction *MDH*, the new optimal growth rate is 0.801. The degree of the flux vector achieving this optimal growth rate now is 4 and the 4-face containing this optimal solution is spanned by 6 EFMs of degree 1. Here there are 4 different decompositions of the optimal solution into 4 EFMs of degree 1.

It should be noted that the optimal solutions to FBA problems are generally not uniquely determined. We chose the solution that was returned by COBRApy. Different solutions with the same optimal value can lie in different faces of the flux cone. Therefore, they can have a different degree and thus also a different number of EFMs in their decomposition.

## The cardinality of minimal metabolic behaviors

We next prove an upper bound on the cardinality of MMBs.

### Proposition 7.1

Let *C* be the flux cone of a metabolic network $\mathcal {N}$ with lineality space *L*. Then for each MMB *D*

$$\begin{aligned} |D |\; \le \; |{{\,\textrm{Irr}\,}}|- (\dim (C) -\dim (L)) +1. \end{aligned}$$

### Proof

By definition of an MMB, there exist a minimal proper face *G* of *C*, $\dim (G) = \dim (L) +1$, and a flux vector $g \in G {\setminus } L$ such that $D = {{\,\mathrm{irr.supp}\,}}(g)$. It follows that *g* is contained in at least $\dim (C) - (\dim (L) + 1)$ facets of *C*. Therefore at least $\dim (C) - ( \dim (L) + 1)$ inequalities in $v_{{{\,\textrm{Irr}\,}}} \ge 0$ are satisfied by *g* with equality, which implies $|{{\,\mathrm{irr.supp}\,}}(g) |= |D |\le |{{\,\textrm{Irr}\,}}|- (\dim (C) - (\dim (L)+1)).$ $\square$

In general, MMBs often contain irreversible reactions for which the non-negativity constraint is redundant. If we remove redundant non-negativity constraints (i.e., shift the corresponding reactions from ${{\,\textrm{Irr}\,}}$ to ${{\,\textrm{Rev}\,}}$) until the description contains no more redundant inequalities, this typically leads to much smaller cardinalities of MMBs.

**Fig. 5:**
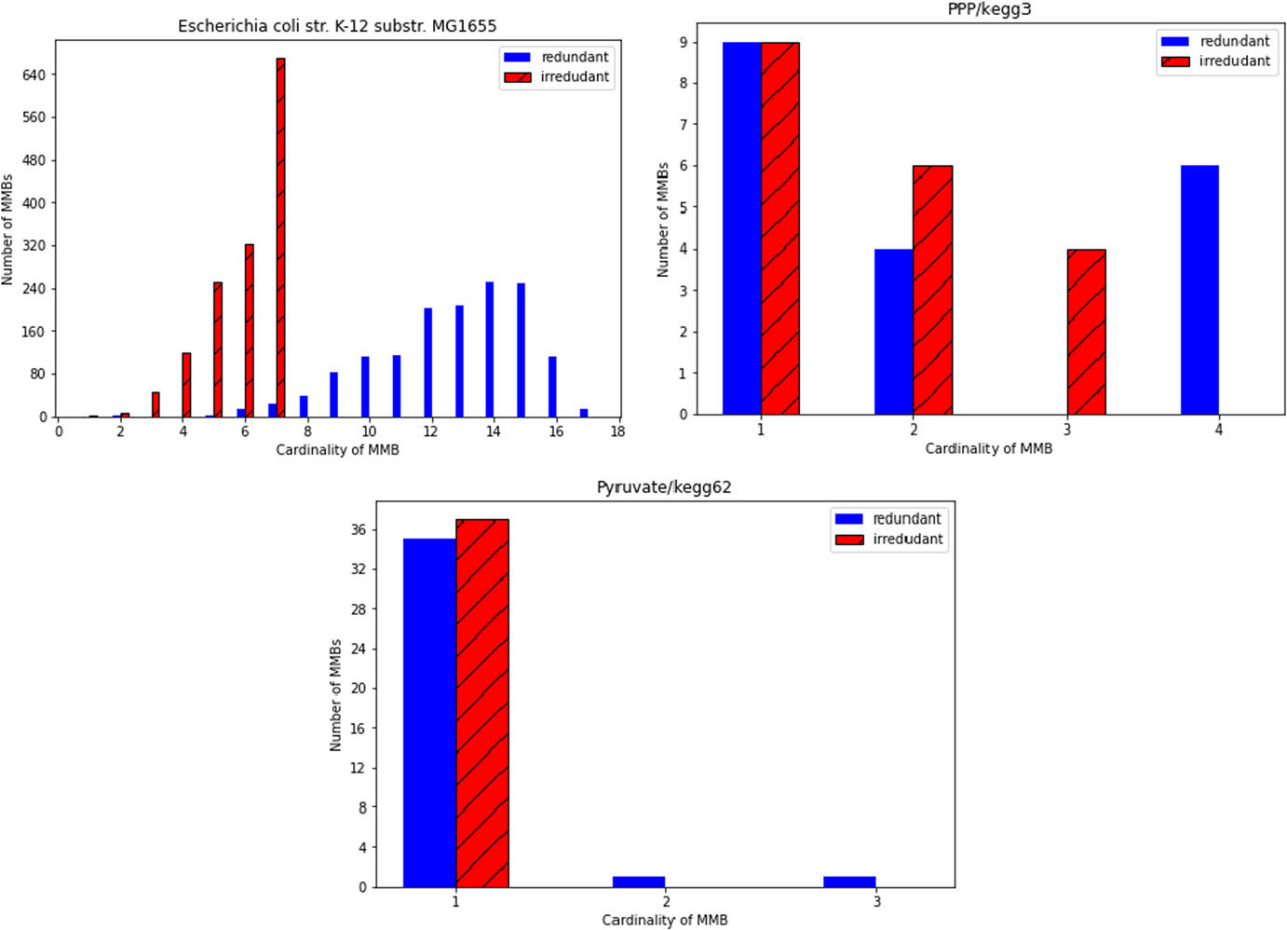
Cardinalities of MMBs with and without redundant irrversibility constraints

For our example networks E.coli core, Pentose Phosphate Pathway and Pyruvate, the number of MMBs is 1421, 19 and 37 respectively. In Fig. 5 we compare the cardinalities of the MMBs in the original description of the flux cone and after removing redundant irreversibility constraints. If we start from the original description, Prop. 7.1 provides the upper bounds 18, 5 and 4 respectively, while the actual maximal sizes of the MMBs are 17, 4 and 3. If we remove redundant non-negativity constraints in lexicographical order (i.e., the redundant non-negativity constraint corresponding to the irreversible reaction with the smallest index is removed first), the bounds become sharp, i.e., we get the bounds 9, 3 and 1 respectively, and these bounds coincide with the actual maximal sizes of the MMBs.

### Proposition 7.2

Let $C = \{ v \in \mathbb {R}^n \mid Sv = 0, v_{{{\,\textrm{Irr}\,}}} \ge 0 \}$ be the flux cone of a metabolic network with lineality space *L* and no redundant inequalities or implicit equalities in $v_{{{\,\textrm{Irr}\,}}} \ge 0$. Then the number of facets of *C* is equal to $\dim (C) - \dim (L)$ if and only if each MMB has cardinality one.

### Proof

By Prop. 3.3, the number of facets is equal to $|{{\,\textrm{Irr}\,}}|$. Thus if $|{{\,\textrm{Irr}\,}}|= \dim (C) - \dim (L)$, then by Prop. 7.1, $1 \le |D |\le |{{\,\textrm{Irr}\,}}|- (\dim (C) - \dim (L)) + 1 = 1$, for each MMB *D* in *C*.

Conversely, if each MMB has cardinality 1, then each minimal proper face is the intersection of all facets of *C* but one. For all $i \in {{\,\textrm{Irr}\,}}$, $G^i = \{v \in \mathbb {R}^n \mid Sv = 0, v_{{{\,\textrm{Irr}\,}}{\setminus } \{i\}} = 0, v_i \ge 0 \}$ is a face of *C*, with $\dim (G^i) \le \dim (L) + 1$. Since $v_i \ge 0$ is not an implicit equality, there exists $g^i \in C$ with $g^i_i > 0$. Let $\bar{D}^i = \{j \in {{\,\textrm{Irr}\,}}\mid g^i_j > 0\}$ be the metabolic behavior defined by $g^i$. By Prop. 4.1, $\bar{D}^i, i \in \bar{D}^i$, is the union of MMBs, which by hypothesis all have cardinality 1. Thus, for all $i \in {{\,\textrm{Irr}\,}}$, $D^i = \{i\}$ is an MMB with the corresponding minimal proper face $G^i$, where $G^i {\setminus } L = \{v \in \mathbb {R}^n \mid Sv = 0, v_{{{\,\textrm{Irr}\,}}{\setminus } \{i\}} = 0, v_i > 0 \}$ and $\dim (G^i) = \dim (L) + 1$. We conclude that the number of minimal proper faces of *C* is equal to the number of facets, which by Prop. 3.3 is equal to $|{{\,\textrm{Irr}\,}}|$.

It remains to prove that $|{{\,\textrm{Irr}\,}}|= \dim (C) - \dim (L)$. Let $U = \{u \in \mathbb {R}^n \mid Su = 0\}$ and $W = \{w \in \mathbb {R}^n \mid w_{{{\,\textrm{Irr}\,}}} = 0\}$. Then $U \cap W = L$ and since by hypothesis there are no implicit equalities, $\dim (C) = \dim (\text {aff}(C)) = \dim (U)$. From the dimension formula, we get $\dim (U+W) = \dim (U) + \dim (W) - \dim (U \cap W) = \dim (C) + (n- |{{\,\textrm{Irr}\,}}|) - \dim (L)$ or $\dim (C) - \dim (L) = |{{\,\textrm{Irr}\,}}|- (n- \dim (U+W))$.

We claim $\dim (U+W) = n$, i.e., $U + W = \mathbb {R}^n$. For each minimal proper face $G^i, i \in {{\,\textrm{Irr}\,}}$, choose $e^i \in G^i {\setminus } L$ with $e^i_i = 1$. Then $e^i_{{{\,\textrm{Irr}\,}}}$ is a unit vector, for all $i \in {{\,\textrm{Irr}\,}}$. Given $v \in \mathbb {R}^n$, let $u = \sum _{i \in {{\,\textrm{Irr}\,}}} v_i \cdot e^i$ and $$w = \begin{pmatrix} v_{{{\,\textrm{Rev}\,}}} - u_{{{\,\textrm{Rev}\,}}} \\ 0 \end{pmatrix}$$. Since $Se^i = 0, i \in {{\,\textrm{Irr}\,}}$, we get $Su = \sum _{i \in {{\,\textrm{Irr}\,}}} v_i \cdot Se^i = 0$ and thus $u \in U$. By definition, $w \in W$. For all $j \in {{\,\textrm{Irr}\,}}$, we have $u_j = \sum _{i \in {{\,\textrm{Irr}\,}}} v_i \cdot e^i_j = v_j e^j_j = v_j$, and thus $u_{{{\,\textrm{Irr}\,}}} = v_{{{\,\textrm{Irr}\,}}}$. Altogether, we get $$u + w = \begin{pmatrix} u_{{{\,\textrm{Rev}\,}}} \\ u_{{{\,\textrm{Irr}\,}}} \end{pmatrix} + \begin{pmatrix} v_{{{\,\textrm{Rev}\,}}} - u_{{{\,\textrm{Rev}\,}}} \\ 0 \end{pmatrix} = \begin{pmatrix} v_{{{\,\textrm{Rev}\,}}} \\ v_{{{\,\textrm{Irr}\,}}} \end{pmatrix} = v$$, which shows $U + W = \mathbb {R}^n$. $\square$

## Conclusion

In this paper, we studied geometric properties of elementary flux modes in metabolic networks. To structure the set of EFMs, we introduce the degree of an EFM and more generally of a flux vector. We show that EFMs of a smaller degree can be seen as more elementary than those of a higher degree, since the EFMs of higher degree can be decomposed into ones of smaller degree. We use the degree of a given flux vector to predict the number of EFMs needed to decompose it and illustrate this with FBA solutions of different degree.

In previous work, see e.g. (Wagner and Urbanczik 2005), EFMs that are not extreme rays of a pointed flux cone were simply considered to be interior rays. We show that EFMs that belong to the (relative) interior of the cone (in the sense of Prop. 5.1) occur only rarely. Our bounds on the degree of EFMs as well as our computational results indicate that the EFMs rather lie in lower-dimensional faces of the flux cone.

Regarding future research, the new insights about the distribution of EFMs in the face lattice of the flux cone raise the question how these can be exploited algorithmically. For example, one could focus on enumerating EFMs of smaller degrees and omit the enumeration of EFMs of larger degrees in order to make EFM analysis for genome-scale metabolic network reconstructions more tractable.

Example 6.5 suggests that FBA solutions may be decomposed into a small number of EFMs. Deepening the understanding of the degree of FBA solutions, different EFM decompositions, and their biological relevance appears to be another promising topic for further research.

In Sect. 7, we analyze the cardinality of minimal metabolic behaviors and study the effect of removing redundant irreversibility constraints. Although irredundant descriptions of flux cones are not uniquely determined, further research is needed to determine the optimal handling of redundant constraints. Some problems benefit from the removal of redundant constraints, while others might benefit from adding them. For example, looking at the faces of the flux cone becomes easier when there are no redundant irreversibility constraints, because then every irreversible reaction corresponds to a facet of the flux cone. Conversely, the reconfiguration method to determine EFMs by splitting reversible reactions may benefit from adding redundant irreversibility constraints. Then fewer reversible reactions have to be split, which reduces the dimension of the reconfigured cone.

## Data Availability

Data was obtained from public domain resources as cited in the paper.
